# The Molecular Bases of the Interaction between a Saponin from the Roots of *Gypsophila paniculata* L. and Model Lipid Membranes

**DOI:** 10.3390/ijms23063397

**Published:** 2022-03-21

**Authors:** Beata Korchowiec, Jacek Korchowiec, Klaudia Kwiecińska, Reneta Gevrenova, Sabine Bouguet-Bonnet, Cheng Deng, Max Henry, Ewa Rogalska

**Affiliations:** 1 Faculty of Chemistry, Jagiellonian University, Ul. Gronostajowa 2, 30-387 Krakow, Poland; korchow@chemia.uj.edu.pl (J.K.); klaudia.kwiecinska1@gmail.com (K.K.); 2 Department of Pharmacognosy, Faculty of Pharmacy, Medical University-Sofia, 2 Dunav Str., 1000 Sofia, Bulgaria; rgevrenova@pharmfac.mu-sofia.bg; 3 Université de Lorraine, CNRS, UMR 7036 CRM2, F-54000 Nancy, France; sabine.bonnet@univ-lorraine.fr (S.B.-B.); chengdengfr@gmail.com (C.D.); max.henry941@gmail.com (M.H.); ewa.rogalska@univ-lorraine.fr (E.R.)

**Keywords:** saponin–membrane interactions, Langmuir films, lipid monolayers, MD simulations, molecular chemistry, membrane models, cholesterol, NMR spectroscopy

## Abstract

In view of the possible medical applications of saponins, the molecular structure of a GOTCAB saponin from the roots of *Gypsophila paniculata* L. was determined by NMR. The biological activity of saponins may depend on the interaction with cell membranes. To obtain more insight in the mechanism of membrane-related saponin function, an experimental and theoretical study was conducted. Ternary lipid systems composed of sphingomyelin, 1-palmitoyl-2-oleoyl-*sn*-glycero-3-phosphocholine, and cholesterol were used as models of mammalian cell membranes. The membrane–saponin interaction was studied experimentally by monitoring surface pressure in the monomolecular films formed at the air–aqueous subphase interface. The behavior of GOTCAB saponin in a water box and model monolayer systems was characterized by molecular dynamics simulations. The results obtained showed that, in the systems used, cholesterol had a decisive effect on the interaction between GOTCAB and phosphocholine or sphingomyelin as well as on its location within the lipid film.

## 1. Introduction

It has recently been shown that saponins may have therapeutic applications [1]. Cytostatic and cytotoxic effects of saponins in the case of malignant tumor cells have been described [2,3]. Other studies have shown that saponins exhibit adjuvant-active properties in vaccines as immunostimulatory complexes [4]. Based on these observations, an open cage-like immunostimulating complex of cholesterol, lipid, immunogen, and saponins from the *Quillaja saponaria* Molina bark (soap bark tree) was used as an active adjuvant in vaccines [5].

An example of a medical application of saponins is Saponinum album (SA; Merck), which is a crude mixture of triterpenoid saponins from *Gypsophila paniculata* L. and *G. arrostii* [6]. SA has been shown to enhance the cytotoxicity of type I ribosome-inactivating protein (RIP-I) saporin from *Saponaria officinalis* L. by 100,000-fold [7]. This interaction between saporin and SA is called “synergistic cytotoxicity principle”. The combination of saporin-based targeted toxins and SA is used in anticancer therapy [8]. If a RIP-I is coupled with specific ligand that targets cancer-associated antigens, the cytotoxicity can even be increased by up to 4,000,000-fold in cell cultures [9]. This represents an important improvement in tumor therapy with targeted toxins as the side effects and costs of such a therapy could be lowered [8]. Böttger et al. [10] analyzed the structure–activity relationship of 56 saponins and their synergistic cytotoxicity with saporin and defined the concept of an ideal saponin. According to this concept, the ideal RIP-I synergistic saponin consists of an oleanane-type aglycone (gypsogenin or quillaic acid); a branched trisaccharide at C-3 consisting of β-d-glucuronic acid, β-d-galactopyranose, and β-d-xylopyranose; and a branched tetrasaccharide at C-28 consisting of deoxy sugars, such as β-d-fucose and/or α-l-rhamnose and acetyl residues.

The *Gypsophila paniculata* L. roots are rich in triterpenoid saponins. They contain about 4% dry weight in the fourth year of ontogenesis of the plant [11]. Phytochemical studies have shown that *G. paniculata* roots and SA comprise glucuronide oleanane-type triterpenoid carboxylic acid 3,28-bidesmosides (GOTCAB saponins) and monodesmosides [12,13,14,15,16,17]. The structure of GOTCAB saponins from *G. paniculata* and *G. arrostii* roots was first reported by Frechet et al. [12]. It was shown that gypsogenin and quillaic acid in the form of aglycones substituted at C-3 with a trisaccharide and with an oligosaccharide via fucose residue at C-28 are present in these molecules. It should be noted that the plant materials used by Frechet et al. were a mixture of both *G. paniculata* and *G. arrostii*. Unfortunately, SA cannot be used for further studies because Merck ceased its production in the 1990s. For the development of a combinatorial anticancer therapy in humans, saponins have to be isolated directly from the roots of *G. paniculata*. For the past 20 years, *Gypsophila* saponins have been purified by chromatographic techniques. A simple method for isolation of *Gypsophila* saponin-1641 from older batches of SA was proposed by Weng et al. [16]. Later, GOTCAB saponins were isolated using chemical degradation of the plant raw material followed by dialysis and HPLC purification [17]. Thakur et al. used an electrophoretic method to isolate saponins from SA [1]. Electrophoresis was also used to separate immune adjuvant saponins from *Quillaja saponaria* [18].

The presence of *Gypsophila* GOTCAB saponins in SA [12,13] was confirmed by Delay et al. [14] and Weng et al. [16,17]. Thus, saponins G1 and G4 [12] were later isolated as *Gypsophila* saponin 1 [17] and SAP030 (*Gypsophila* saponin 2) [14,17].

Different studies conducted by our group concerning *Gypsophila* species [19,20,21] have been previously published. Here, we present the results of a work aimed at elucidating the structure of a GOTCAB saponin from *G. paniculata* roots. This structural study was performed using NMR analysis. The molecular structure obtained was a starting point for further work concerning the role of saponins in pharmaceutical preparations. Because the interaction with cell membranes may play a role in the therapeutic activity of saponins, an experimental study was conducted using model mammalian cell membranes and molecular modeling.

## 2. Results and Discussion

### 2.1. NMR Analysis

1D and 2D NMR techniques were performed in order to elucidate the chemical structure of the isolated GOTCAB saponin (Supplementary Materials, Figure S2). The proton and carbon resonances of the aglycone moiety were assigned from the analysis of ^1^H–^1^H COSY, HSQC and HMBC spectra, the aglycone was identified as quillaic acid by comparison with literature data [14]. The ^1^H NMR spectrum showed signals ascribable to six tertiary methyl groups at δ 0.72, 0.78, 0.83, 1.20, 1.50 ppm. Also evident were signals of H-23 at 9.55 ppm due to the aldehyde function at C-4 and of H-16 at δ 4.85 ppm due to the presence of a hydroxyl group at C-16. Complete assignment of the aglycone moiety is given in Table 1.

**Figure 1:**
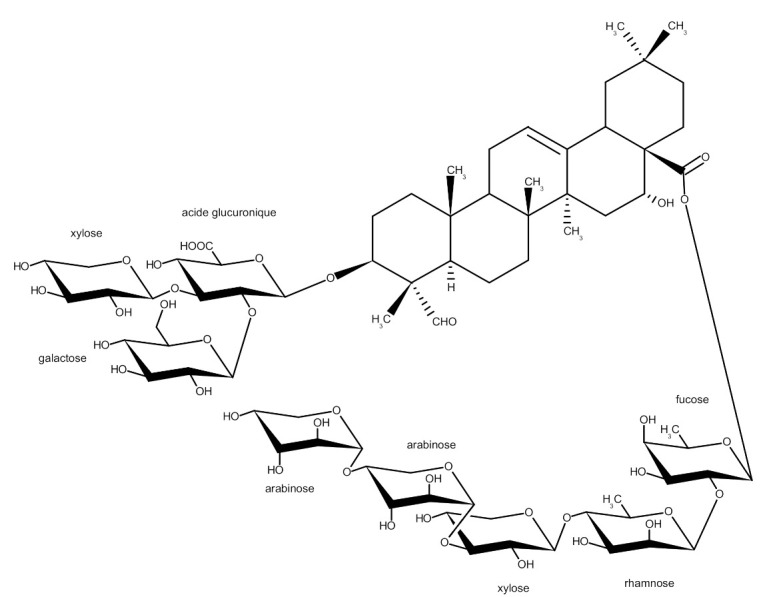
Schematic view of the structure of the GOTCAB saponin from the roots of *Gypsophila paniculata* L. as determined by NMR.

For the sugar moiety, eight anomeric protons at δ 5.59 ppm (d, *J* = 8.4 Hz), 4.89 ppm (d, *J* = 7.7 Hz), 4.70 (d, *J* = 7.7 Hz), 5.04 (d, *J* = 7.7 Hz), 4.85 (d, *J* = 7.7 Hz), 5.82 (d, *J* = 1.1 Hz), 5.16 (d, *J* = 7.5 Hz), and 4.66 (d, *J* = 7.1 Hz) were observed. Complete assignment of each sugar resonances was achieved by considering the HSQC-TOCSY and ^1^H–^1^H COSY spectra. According to spin–spin couplings and chemical shifts [12,14], one β-fucose (Fuc, Fuc-H1 at 5.59 ppm), two α-arabinose (Ara, Ara-H1 at 4.89 ppm; Ara’, Ara’-H1 at 4.70 ppm), two β-xylose (Xyl, Xyl-H1 at 5.04 ppm; Xyl’, Xyl’-H1 at 4.85 ppm), one α-rhamnose (Rha, Rha-H1 at 5.82 ppm), one β-galactose (Gal, Gal-H1 at 5.16 ppm), and one β-glucuronic acid (GlcA, GlcA-H1 at 4.66 ppm) were identified (assignments in Table 2). Glycosidation shifts were observed for Fuc-C2 (δ 73.4 ppm), Ara-C4 (δ 77.8 ppm), Xyl’-C3 (δ 84.6 ppm), Rha-C4 (δ 82.7 ppm), GlcA-C2 (δ 76.6 ppm), and GlcA-C3 (δ 84.2 ppm). The cross-peak in the HMBC experiment between GlcA-H1 (δ 4.66 ppm) and C-3 of the aglycone (δ 84.4 ppm) showed that the glucuronic acid was linked to the aglycone at C-3 position. Chemical shifts of Fuc-H1 (δ 5.59 ppm) and Fuc-C1 (δ 93.7 ppm) as well as the HMBC cross-peak between Fuc-H_1_ (δ 5.59 ppm) and C-28 of the aglycone (δ 175.8 ppm) suggested that this sugar was involved in an ester linkage with the C-28 carboxylic group of the aglycone moiety. Key HMBC correlations were used in order to determine the position and the sequence of the sugar residues: between the proton signal at δ 5.16 ppm (Gal-H1) and the carbon resonance at δ 76.6 ppm (GlcA-C2), between the proton signal at δ 5.04 ppm (Xyl-H1) and the carbon resonance at δ 84.2 ppm (GlcA-C3), between the proton signal at δ 5.82 ppm (Rha-H1) and the carbon resonance at δ 73.4 ppm (Fuc-C2), between the proton signal at δ 4.85 ppm (Xyl’-H1) and the carbon resonance at δ 82.7 ppm (Rha-C4), between the proton signal at δ 4.89 ppm (Ara-H1) and the carbon resonance at δ 84.6 ppm (Xyl’-C3), and between the proton signal at δ 4.70 ppm (Ara’-H1) and the carbon resonance at δ 77.8 ppm (Ara-C4). The saponin was finally identified as corresponding to the structure presented in Figure 1.

It should be noted that extensive analysis of the NMR spectra described above allowed us to detect the presence of another saponin in the sample but at a very low concentration compared to other molecule described here (more than 5 times less according to the NMR signals integrals). Due to this low concentration, it was possible to distinguish the NMR signals of the two molecules, but it was not possible to completely assign the second one. Nevertheless, it can be assumed than this molecule was very similar to the GOTCAB saponin but had a gypsogenin moiety instead of quillaic acid and a different sequence of eight sugar moieties.

The structure obtained in this work (Figure 1) was a starting point for further study on the GOTCAB–lipid membrane interaction.

### 2.2. Compression Isotherms

The interactions between GOTCAB saponin and the model cell membranes were investigated experimentally using Langmuir film technique [22,23,24]. The monomolecular films were formed with ternary SM/POPC/CHOL lipid mixtures containing biologically relevant 10 or 30% of cholesterol [25,26]; films containing 50 mol% of cholesterol were used as well. The SM/POPC equimolar ratio was kept constant. The final composition of ternary SM/POPC/CHOL monolayers was 45/45/10, 35/35/30, and 25/25/50. Some saponins show a high affinity for cholesterol, which is often linked to their hemolytic activity [27,28,29]. However, it was recently shown that membrane activity of the ginsenoside Rh2 saponin was boosted by the interaction with sphingomyelin but was worsened by cholesterol [30]. The membrane models used in this study were composed of a saturated (SM) and unsaturated (POPC) phospholipid as well as cholesterol; the latter is found in the raft structures of biological membranes. Sphingomyelin and cholesterol are involved in the formation of a raft-like liquid-ordered (L_o_) phase, while POPC is involved in the nonraft liquid-disordered (L_d_) phase [31,32,33]. 

**Figure 2:**
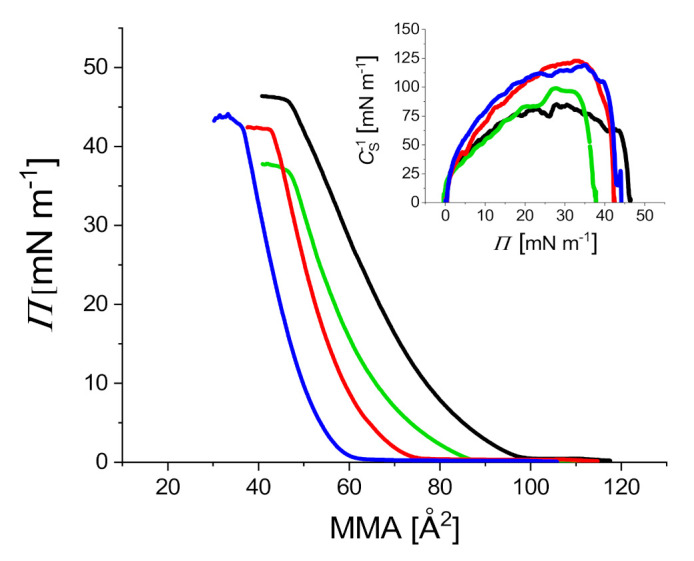
Compression isotherms of SM/POPC (black), SM/POPC/CHOL 10 mol% (green), SM/POPC/CHOL 30 mol% (red), and SM/POPC/CHOL 50 mol% (blue) mixed films spread on the water subphase. Inset: *C*_S_^−1^–*Π* dependency. *T* = 20 °C.

The monomolecular films formed with ternary SM/POPC/CHOL mixtures on the pure water or aqueous GOTCAB subphases were characterized using compression isotherms. The interactions of GOTCAB with the model membranes were monitored by measuring the surface pressure (*Π*) as a function of molecular area (*A*). As a reference, a cholesterol-free equimolar SM/POPC monolayer was used. The isotherms obtained on the pure water subphase are presented in Figure 2. With increasing concentration of CHOL in the film, the isotherms shifted to lower molecular areas and the film rigidity increased, as indicated by the compression modulus (Figure 2, the inset). This outcome agrees with many results published in the literature concerning phospholipid films containing cholesterol [34,35,36,37,38].

**Figure 3:**
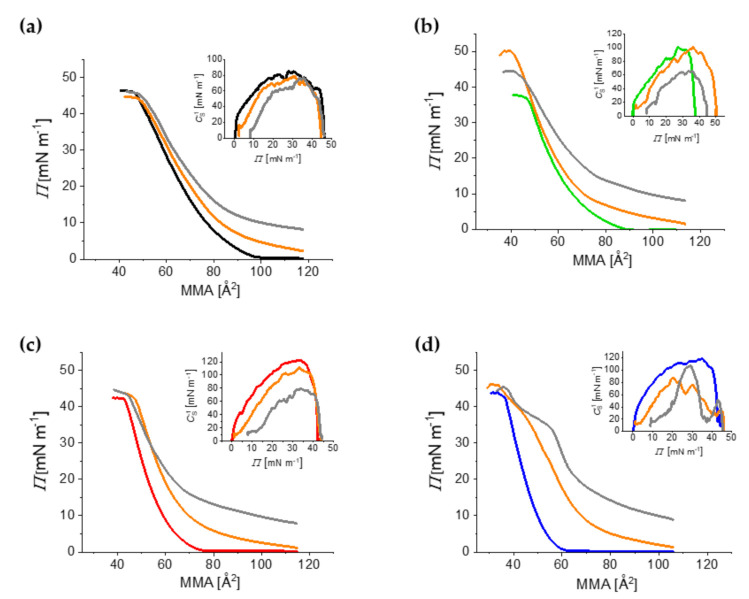
*Π*–*A* isotherms of SM/POPC (**a**), SM/POPC/CHOL 10 mol% (**b**), SM/POPC/CHOL 30 mol% (**c**), and SM/POPC/CHOL 50 mol% (**d**) spread on the water subphase (black (**a**), green (**b**), red (**c**) and blue (**d**)) and the GOTCAB solution subphase with 8 (orange (**a**–**d**)) and 80 (gray (**a**–**d**)) mg L^−1^ GOTCAB. *T* = 20 °C. Insets: *C*_S_^−1^–*Π* dependency.

In the case of the subphases containing GOTCAB, the isotherms shifted to higher molecular areas compared to pure water. This effect indicates the interaction between GOTCAB and the monolayers. As in the case of the pure water subphase, the SM/POPC/CHOL and SM/POPC films had different properties as well (Figure 3). Indeed, the isotherms of the films containing cholesterol shifted to lower molecular areas compared to the binary films. In general, the films containing cholesterol were more rigid compared to the binary films (Figure 3 insets and Figure S3 of Supplementary Materials).

However, the ternary SM/POPC/CHOL 50 mol% films spread on GOTCAB were less stable compared to pure water, as indicated by a lower surface pressure at the collapse (*Π*_coll_) of the monolayer (Figure 3). This effect was particularly well seen in the case of 80 mg·L^−1^ GOTCAB.

The *Π*_coll_ values of SM/POPC/CHOL 50 mol% were around 35 and 29 mN·m^−1^ on water and 80 mg·L^−1^ GOTCAB, respectively. These observations indicate that cholesterol modifies the molecular interaction between GOTCAB and film-forming lipids. It may be supposed that GOTCAB forms different molecular associations with lipids depending on the presence of cholesterol in the film.

### 2.3. Molecular Dynamics Simulations

To obtain a better understanding of the experimental measurements, molecular dynamics simulations were used [39,40,41,42], which allowed the properties of the model membranes and molecular interaction within the system containing GOTCAB molecules to be studied at an atomic level.

#### 2.3.1. Saponin Molecules in a Box of Water

**Figure 4:**
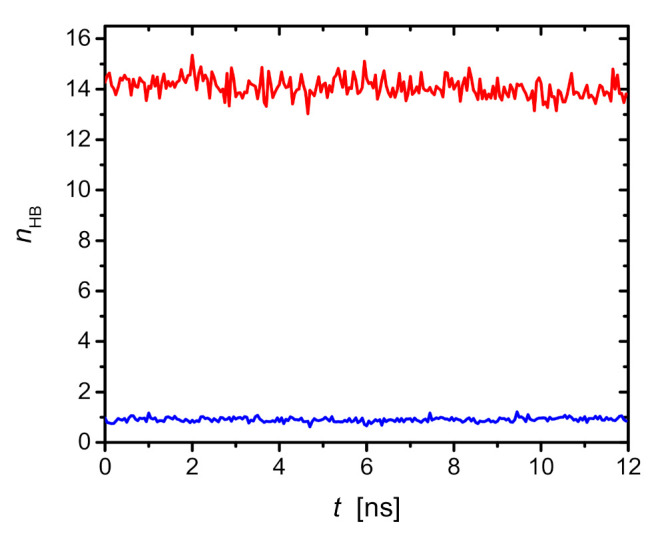
Average number of hydrogen bonds formed by one GOTCAB molecule as a function of time. The results are averaged over three independent simulations performed for GOTCAB/H_2_O systems. The red curve corresponds to HB formed by the GOTCAB and water molecules, while the blue curve corresponds to intra- or inter-GOTCAB HBs.

Figure 4 shows the average number of hydrogen bonds (HB), $n_{HB}$, formed by saponin with water molecules and the average number of hydrogen bonds between saponin molecules.

As can be seen, GOTCAB HBs were formed preferentially with water. Approximately one GOTCAB molecule was involved in 14 HBs of water molecules. On the other hand, the number of HBs among GOTCAB molecules was less than one. Detailed analysis of the data obtained showed that intramolecular HBs were more common. The latter indicates that HBs are not responsible for the clustering the GOTCAB molecules. 

The formation of the GOTCAB cluster is summarized in Table 3. The results correspond to a 12 ns production run. The numbers given are equal to the average number of clusters of a given size $\langle N_{{\left(GOT\right)}_{n}}\rangle$ observed during the last runs, where (GOTCAB)*_n_* is a cluster built of *n* monomers, and n=1,2,… denotes monomers, dimers, and so on. The numbers in each row are normalized to the total number of saponin molecules in the simulation box (27 in systems I–III): $\sum_{n}n\cdot \langle N_{{\left(GOT\right)}_{n}}\rangle =27$. For example, the average number of monomers, dimers, etc. in system I at a given time was equal to 7.39, 4.69, etc., respectively. The same was true for small clusters, namely dimers and trimers. Larger clusters, with the exception of the tetramer for system III, were observed temporarily. For example, 0.58 for system II and $N_{\mathrm{GOTCAB}}=7$ means a cluster was observed for almost 7 ns (0.58 × 12 ns ≈ 7 ns). This does not mean, however, that it is also the lifetime of such a cluster. A single cluster of this size was stable for 3 ns during the production run. Clusters of at least 9 monomers were not observed. The obtained data show that GOTCAB is present in water in the form of monomers and small aggregates. It has to be mentioned that the results obtained are qualitative and show a tendency.

**Figure 5:**
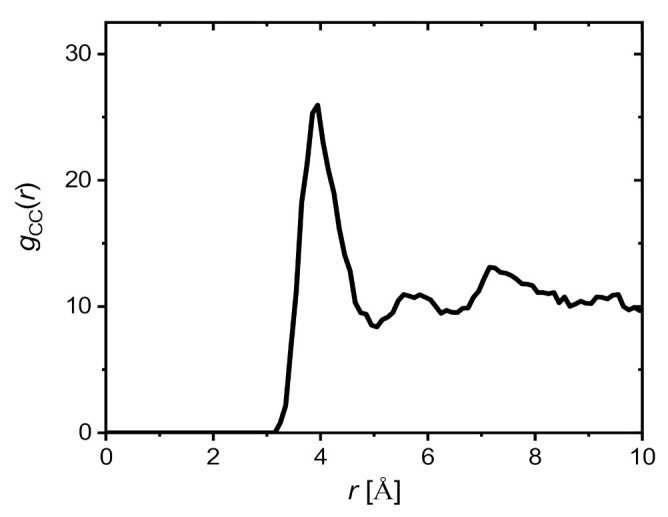
Radial pair distribution function for methyl carbon atoms of the aglycone moiety and methyl carbon atoms of rhamnose and fucose residues. The presented curve is an average over three independent simulations (system I–III, see Table 2).

**Figure 6:**
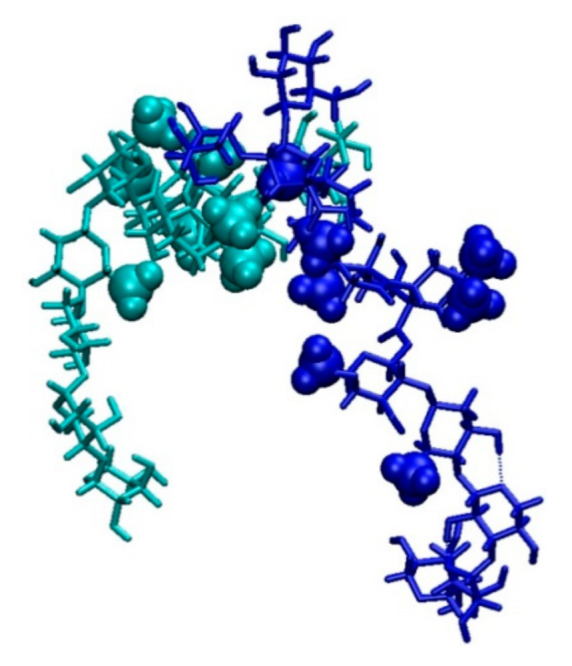
GOTCAB saponin dimer with monomers shown in blue and cyan, respectively. The aglycone CH_3_ groups are represented by balls dimensioned in proportion to the van der Waals radii. The dashed line represents an intramolecular HB.

The HB pattern shown in Figure 4 indicates a hydrophobic origin of clustering. The structure of GOTCAB saponin shows many CH_3_ groups attached to different residues (rhamnose, fucose, and aglycone). It can be expected that in clusters, such groups will be closer to each other. Therefore, a conformation with closely packed nearby CH_3_ substituents will be preferred in the monomer. Such a spatial arrangement will minimize unfavorable interaction with the solvent. Figure 5 shows the radial pair distribution function for aglycone CH_3_ as well as rhamnose and fucose. The first maximum at approximately 4 Å is a clear indicator of this preferred arrangement of the methyl groups. This situation is illustrated for the GOTCAB dimer in Figure 6.

#### 2.3.2. Saponin Molecules at the Air–Water Interface

**Figure 7:**
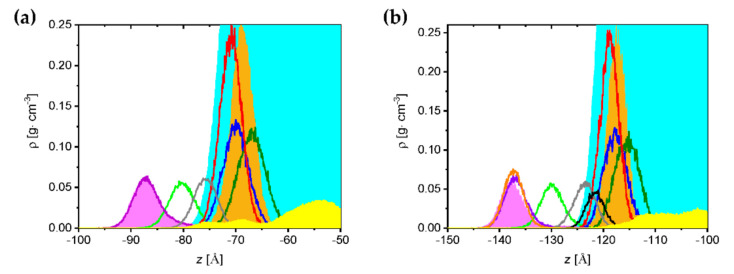
Partial density plots at the interface of SM/POPC (**a**) and SM/POPC/CHOL 30 mol% (**b**) monolayers interacting with GOTCAB. Color code; cyan area: water; magenta area: terminal CH_3_ in the POPC lateral chains; violet line: terminal CH_3_ in the SM lateral chains; orange line: terminal CH_3_ in the CHOL lateral chain; green line: C=C in POPC; gray line: C=C in SM; orange area: PO_4_ in POPC; red line: PO_4_ in SM; black line: OH in CHOL; olive line: N(CH_3_)_3_ in POPC; blue line: N(CH_3_)_3_ in SM; yellow area: carbon atoms of the aglycone moiety in GOTCAB.

Partial density plots along the *z* axis, i.e., in the direction perpendicular to the monolayer surface, are shown in Figure 7. Panels (a) and (b) correspond to binary SM/POPC and ternary SM/POPC/CHOL 30 mol% monolayers in the presence of GOTCAB molecules, respectively.

Figure 7 illustrates densities of the terminal hydrophobic CH_3_ groups in phospholipid lateral chains, hydrophilic PO_4_, N(CH_3_)_3_, and OH functional groups. Moreover, the location of C=C double bonds is shown. Independently of the system, the outspread of the hydrophobic parts of the lipids towards vacuum was almost the same. The maximum of the partial density plot of terminal CH_3_ groups of POPC (magenta area) and SM (violet line) in panel (a) were located in the same region, including the location of maxima. The same was true for the ternary system. An additional orange line representing the terminal CH_3_ groups of CHOL was exactly in the same place as the remaining two lines. The differences were more pronounced in the hydrophilic region. Here, the partial density plots of PO_4_ in POPC (orange area in both panels) shifted towards the water box interior compared to the SM PO_4_ groups (red line). The hydrophobic part of CHOL, represented by the black line in panel (b), was located at the interface. The same behavior was found in POPC N(CH_3_)_3_ and SM N(CH_3_)_3_ groups, with the former pushed further towards the water bulk. A similar trend was observed in the SM/POPC and SM/POPC/CHOL monolayers formed on pure water (Supplementary Materials, Figure S4).

In Figure 7, the interaction of GOTCAB and SM/POPC or SM/POPC/CHOL is indicated by the relative positions of the corresponding maxima. It can be observed that the yellow areas (GOTCAB) presented in panels (a) and (b) are dissimilar. In the binary system (panel a), GOTCABs interacted with the hydrophilic and partially hydrophobic regions of the monolayer formed by different lipid moieties. In the ternary monolayer (b), the yellow area shifted towards the aqueous subphase, indicating that polar interactions between GOTCABs and film-forming lipids were favored. These results show that in the presence of cholesterol, GOTCABs do not penetrate into the monolayer. 

**Figure 8:**
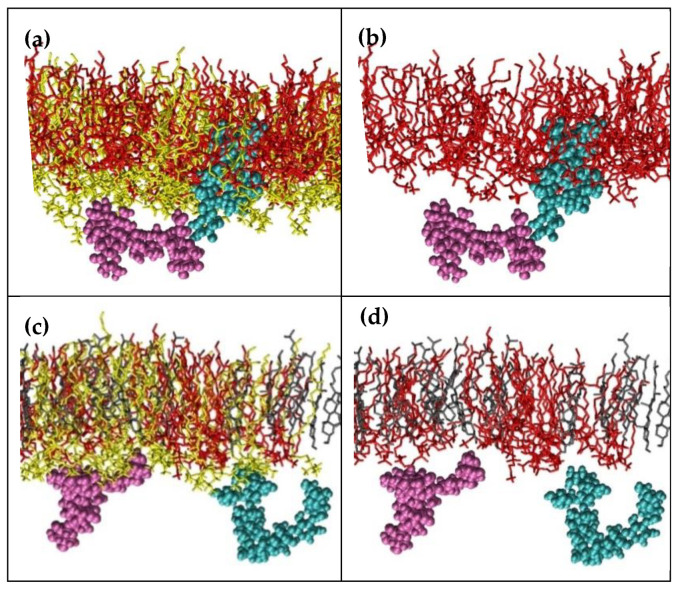
Side views of SM/POPC (**a**,**b**) and SM/POPC/CHOL 30 mol% (**c**,**d**) monolayers interacting with GOTCAB molecules, the last frame of production run trajectories. Panels (**a**,**c**): POPC shown; (**b**,**d**): POPC not displayed. Aliphatic hydrogens are removed for clarity. Color code: SM: red, CHOL: gray, POPC: yellow, GOTCAB: for more clarity, the two identical molecules are presented in cyan and mauve.

The side view of the monolayers is presented in Figure 8. In panels (a) and (c), all molecules forming the monolayer are shown; in panels (b) and (d), POPC molecules are not displayed to facilitate visualization of the interaction between GOTCAB and the lipids. It can be observed that in the binary SM/POPC system, GOTCABs formed aggregates in the aqueous phase (see Figure 5), while SM–GOTCAB complexes were formed via polar and apolar interactions in the film (Figure 8a,b). In the case of GOTCABs immersed in water, the small number of hydrogen bonds was due to interactions between sugar OH groups and oxygen atoms in the phospholipid PO_4_ groups. However, most HBs were formed with water molecules. In the SM–GOTCAB complexes, the aglycone moieties interacted with the SM hydrocarbon chains, while the sugar residues were present in the hydrophilic region of the SM/POPC film. The results obtained showed that –NH– and OH groups in SM were involved in the reorientation of GOTCABs in the monolayer (Figure S5). Indeed, sugar and aglycone hydroxyl oxygens formed hydrogen bonds with nitrogen and oxygen atoms of the –NH–CO– group, respectively, stabilizing GOTCAB in the apolar region of the lipid film (Figures S5 and S6). The –O–CO– groups in POPC were practically not involved in hydrogen bond formation (compare both curves in Figure S5). It can be noted that the effects observed resemble the flip/flop mechanism described in the case of the CX1 calixarene derivative [40,43].

In the ternary SM/POPC/CHOL system, GOTCAB cluster distribution resembled that observed in pure water (compare Figure 5 and red curve of Figure S7). Here, GOTCABs interacted with the POPC polar heads and stayed at the lipid–aqueous interface (Figure 8c,d). The latter observation was in accordance with that based on the density profile analysis (Figure 7). Overall, the results obtained with modeling indicate that cholesterol hinders penetration of GOTCABs into the monolayer and makes formation of GOTCAB–SM complexes less likely. This observation is consistent with some results published in the literature showing that, under the conditions used, cholesterol is preferentially associated with sphingomyelin [44].

## 3. Materials and Methods

### 3.1. Materials

The saponin used in this study was purified as described previously [21]. The molecular structure of the GOTCAB saponin is shown in Figure 1. Sphingomyelin (Egg SM, ~99.9% pure) and 1-palmitoyl-2-oleoyl-*sn*-glycero-3-phosphocholine (POPC, ~99.9% purity) were from Avanti Polar Lipids. Cholesterol (CHOL) and chloroform (~99.9% purity) used for preparing phospholipid solutions were from Sigma-Aldrich. Pure water and aqueous GOTCAB solutions with concentrations of 8 and 80 mg·L^−1^, respectively, were used as subphases for the monolayer experiments. The subphases were prepared with MilliQ water that had a resistivity of 18.2 MΩ·cm at 25 °C, surface tension of 72.8 mN·m^−1^ at 20 °C, and pH of 5.6.

### 3.2. NMR Experiments

NMR experiments were performed on a Bruker Avance III spectrometer operating at 9.4 Tesla (400 Mhz and 100.6 MHz for ^1^H and ^13^C, respectively) using a Bruker 5 mm BBFO probe. Pulse widths were 14.1 and 10.5 μs for ^1^H and ^13^C, respectively. Samples were dissolved in 400 μL of 50% pyridine-d_5_ and 50% D_2_O (pyridine-d_5_ was used as reference, with highest field signals at 7.19 ppm for ^1^H and 123.5 ppm for ^13^C), and all experiments were performed at 313 K. Standard experiments were performed using the Bruker software package: ^1^H, ^13^C decoupled from proton, ^13^C JMOD, ^1^H–^1^H DQF-COSY, ^1^H–^13^C HSQC, ^1^H–^13^C HMBC, and ^1^H–^13^C HSQC-TOCSY.

Acquisition parameters: ^1^H spectral width 4400 Hz, ^13^C spectral width 24,000 Hz, repetition time 2 s. Double Quantum Filtered COSY experiment: 4K complex points × 512 increments, 16 scans per increment. HSQC experiment: 2K × 512 data set, 128 scans per increment. HMBC experiment: a typical value of 50 ms was used for the evolution of long-range coupling and a value of 3.4 ms for the low-pass *J* filter; 2K × 512 data set, 128 scans per increment. HSQC-TOCSY experiment: DIPSI2 scheme for homonuclear Hartman–Hahn mixing (80 ms), 2K × 512 data set, 128 scans per increment.

### 3.3. Surface Pressure–Area Isotherms

The surface pressure (*Π*) measurement was carried out using a KSV 2000 Langmuir balance (KSV Instruments, Helsinki, Finland). A Teflon trough (58 × 15 × 1 cm) with two hydrophilic Delrin barriers providing a symmetric compression was used in all experiments. Surface pressure measurements were carried out using the Wilhelmy plate method. The apparatus was closed in a Plexiglas box, and the temperature was kept constant at 20 °C. All impurities were removed from the subphase surface by sweeping and suction. Monolayers were spread from chloroform solutions of accurate lipid concentrations using a microsyringe (Hamilton Co., Reno, NV, USA). SM/POPC mole fraction was 0.5 (50 mol%). CHOL mole fraction in the SM/POPC/CHOL mixtures was 0.1, 0.3, or 0.5 (10, 30, or 50 mol%, respectively).

After the equilibration time of 20 min, the films were compressed at the rate of 2.5 mm∙min^−1^ by two symmetrically moving barriers. A PC computer and KSV software were used to control the experiments. Each compression isotherm was performed at least three times. The accuracy of the results was ±0.1 Å^2^ for mean molecular area and ±0.01 mN·m^−1^ for surface pressure measurements.

The surface pressure compression isotherms (*Π*–*A*) allowed the compressibility modulus to be determined (*C*_S_^−1^ = −*A*(d*Π*/d*A*)*_T_*) [45,46,47]. 

### 3.4. Molecular Dynamics Simulations

**Figure 9:**
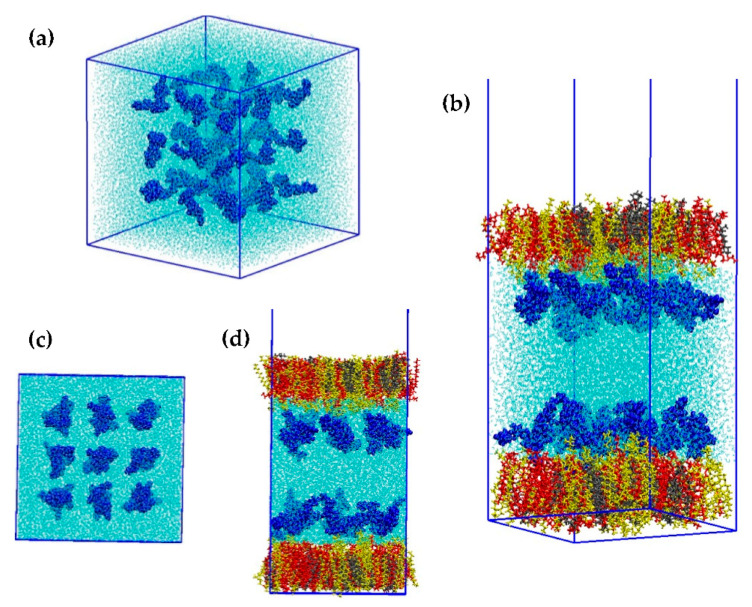
Perspective (**a**,**b**) and side (**c**,**d**) views of initial configurations of GOTCAB/H_2_O (**a**,**c**) and SM/POPC/CHOL/GOTCAB (**b**,**d**) molecular systems. Color code: GOTCAB: blue, SM: red, CHOL: gray, POPC: yellow, water: cyan.

The behavior of GOTCAB saponin in a water box and model monolayer systems was characterized by molecular dynamics (MD) simulations. The former was composed of 27 GOTCAB molecules distributed randomly on 3 × 3 × 3 grid. In the symmetric monolayer system, 9 GOTCAB molecules were placed 10 Å under/above the equilibrated monolayers on 3 × 3 grid. Two model monolayers were built: a binary monolayer comprising SM and POPC (SM/POPC monolayer) and a ternary monolayer with addition of CHOL molecules (SM/POPC/CHOL monolayer). The initial configurations for both types of simulations are shown in Figure 9. The GOTCAB saponin in the water box is shown in panels a and c. The ternary monolayer model with GOTCAB is shown in panels b and d. All-atom CHARMM force field was applied [48,49]. The missing parameters of GOTCAB saponin were derived using the force field toolkit of VMD graphical software [50,51]. The initial structure of GOTCAB, namely one of the many conformers, was optimized at the B3LYP/6-31G(d) level of theory. Due to its size, the saponin was divided into smaller fragments, each retaining the chemical identity of the immediate environment. A force field toolkit was applied to each fragment separately. Such a procedure is in line with the standard assumption of molecular dynamics sumulations, i.e., the transferability of force field parameters. The parameters derived in this way were combined together, including the charge distribution.

The MD calculations were carried out using the NAMD package [52] with periodic boundary conditions imposed on the system. Langevin thermostat and barostat were used to control the temperature and pressure, respectively. van der Waals interactions were switched off at 12 Å. Electrostatic energy was calculated with the PME method [53]. A time step of 1 fs was used in all simulations. TIP3P model was adopted for water [54]. The VMD software was also applied to visualize the trajectories and to compute selected properties, including hydrogen bond characteristics. It was assumed that the distance from the hydrogen donor (HD) atom to the hydrogen acceptor (HA) atom was less than or equal to 3 Å and that the HD-H-HA angle was not greater than 20 degrees. 

In all GOTCAB/H_2_O calculations, the number of atoms (*N*) in the simulation box was greater than 160,000. Three independent simulations, each of 120 ns, were performed for GOTCAB saponins. The systems were equilibrated in (*N*, *p*, *T*) ensembles for 60 ns with *T* = 293 and *p* = 1 bar. The second half of calculations were performed in canonical ensembles. The last 12 ns runs were used to compute the average quantities. The binary and ternary monolayers consisted of 100 lipid molecules. The number of atoms in all systems were greater than 120,000. 

Monolayers were placed on two sides of a water slab (*xy* cut). After the initial 10 ns canonical ensemble simulations, the calculations were carried out at constant temperature (*T* = 293 K), normal pressure (*p*_n_ = 1 bar), and surface tension. Two values of surface tension (*γ* = 50 and 30 mN·m^−1^) were considered. The (*N*, *T*, *p*_n_, *γ*) simulations were performed for 120 ns. These calculations were followed by 60 ns canonical ensemble calculations with the box size fixed from (*N*, *T*, *p*_n_, *γ*) calculations (average from the last 12 ns). Thus, the total simulation time for each system was 190 ns. The data presented in the paper were computed using the last 24 ns of the final canonical ensemble run being considered as the production run. The picture presenting the equilibration process is shown in Figure S1 in the Supplementary Materials (after 100 ns, the total potential energy fluctuated around the average value).

## 4. Conclusions

The results obtained in this work clarify the mechanism of the interaction between a pure GOTCAB saponin with defined molecular structure and model cell membranes containing phospholipids or phospholipids and cholesterol. In the monolayers prepared with the two phospholipids, namely sphingomyelin and phosphatidylcholine, GOTCAB established polar interaction with the phospholipid polar heads (PO_4_ groups) via sugar moieties (OH groups), and apolar interaction between the aglycone moiety and hydrocarbon chains present in sphingomyelin. Obviously, the interaction with the latter was favored compared to the phosphatidylcholine chains. The conjunction of polar and apolar interactions allowed stabilization of the GOTCAB in the apolar part of the monolayer. In the ternary monolayers, the interaction between cholesterol and sphingomyelin was favored compared to GOTCAB–sphingomyelin. This effect may be explained by a better cholesterol match between the hydrocarbon chains compared to the saponin aglycone moiety. Consequently, in ternary films, GOTCAB was stabilized in the region of the lipid polar heads. It can be concluded that, in view of its effectiveness, competition with cell membrane cholesterol has to be considered when medical, saponin-based preparations are developed. The structure and purity of the saponin used may be crucial for the final biological effect of such preparations.

## Figures and Tables

**Table 1:** ^1^H and ^13^C data (ppm) for the aglycone moiety in GOTCAB; 1:1 pyridine_d5/D_2_O.

Atom Number	^1^H (ppm) (J/Hz)	^13^C (ppm)
1	1.39; 0.78	37.4
2	1.83; 2.13	23.8
3	3.90	84.4
4	–	54.8
5	1.22	43.7
6	0.85; 1.22	19.7
7	1.31; 1.46	31.7
8	–	39.2
9	1.60	46.0
10	–	35.3
11	1.60; 1.74	22.7
12	5.38	121.7
13	–	143.2
14	–	41.0
15	1.72; 1.89	35.2
16	4.85	72.9
17	–	48.3
18	3.12	40.9
19	1.07; 2.41	46.3
20	–	29.7
21	2.02; 1.12	34.7
22	2.13; 1.90	30.3
23	9.55	210.5
24	1.20	9.5
25	0.72	14.8
26	0.83	16.5
27	1.50	26.2
28	–	175.8
29	0.78	32.1
30	0.78	23.6

**Table 2:** ^1^H and ^13^C data (ppm) for the sugar moieties in GOTCAB; 1:1 pyridine_d5/D_2_O.

Atom Number	^1^H (ppm) (J/Hz)	^13^C (ppm)
Fucose (Fuc)		
1	5.59 (d, 8.4)	93.7
2	4.25 (t, 8.8)	73.4
3	3.90 (m)	74.6
4	3.90 (m)	71.5
5	3.75 (m)	71.5
6	1.31 (d, 6.3)	15.5
Arabinose (Ara)		
1	4.89 (d, 7.7)	103.6
2	4.13 (t, 8.3)	71.5
3	4.01 (m)	72.4
4	4.17 (m)	77.8
5	4.33 (dd, 12.7, 2.3) 3.73 (m)	65.3
terminal Arabinose (Ara’)		
1	4.70 (d, 7.7)	105.1
2	4.07 (t, 7.6)	71.1
3	3.90 (m)	72.4
4	4.09 (m)	68.0
5	4.05 (dd, 9.6, -) 3.66 (dd, 9.6, -)	65.9
terminal Xylose (Xyl)		
1	5.04 (d, 7.7)	103.0
2	3.67 (t, 8.2)	73.7
3	3.86 (m)	76.2
4	3.89 (m)	69.4
5	4.17 (dd, 10.1, 5.0) 3.55 (t, 10.1)	65.0
Xylose (Xyl’)		
1	4.85 (d, 7.7)	105.1
2	3.72 (t, 8.4)	73.9
3	3.87 (m)	84.6
4	3.81 (m)	67.7
5	3.98 (dd, 11.1, 5.6) 3.38 (t, 11.1)	65.2
Rhamnose (Rha)		
1	5.82 (d, 1.1)	100.1
2	4.46 (d, 3.3)	70.3
3	4.26 (dd, 9.1, 3.3)	70.8
4	3.98 (m)	82.7
5	4.10 (m)	67.6
6	1.49 (d, 6.2)	17.3
terminal Galactose (Gal)		
1	5.16 (d, 7.5)	102.3
2	3.97 (dd, 7.5, 9.3)	71.7
3	3.92 (m)	73.2
4	4.18 (m)	69.1
5	3.8 (m)	75.4
6	4.16 (m) 4.02 (m)	61.1
Glucuronic acid (GlcA)		
1	4.66 (d, 7.11)	102.4
2	4.10 (m)	76.6
3	4.10 (m)	84.2
4	4.00 (m)	70.5
5	4.08 (m)	76.8
6	-	174.7

**Table 3:** Average number of clusters of GOTCAB molecules, $\langle N_{{\left(GOT\right)}_{n}}\rangle$, during the 12 ns production run trajectories.

	*n* = 1	2	3	4	5	6	7	8
System I	7.39	4.69	1.82	0.26	0.44	0.16	0.08	0.00
System II	9.58	2.04	1.52	0.32	0.47	0.15	0.58	0.03
System III	8.69	2.40	1.07	1.75	0.35	0.20	0.05	0.00

## Supplementary Materials

The following supporting information can be downloaded at: https://www.mdpi.com/article/10.3390/ijms23063397/s1.
