# Supplementary material for: The Molecular Bases of the Interaction between a Saponin from the Roots of Gypsophila paniculata L. and Model Lipid Membranes
Source: Int J Mol Sci. 2022 Mar 21;23(6):3397. doi: 10.3390/ijms23063397 (PMC8949875; doi:10.3390/ijms23063397)
Supplement: Supplementary file 1 [file ijms-23-03397-s001.zip › Supplementary Materials.pdf]

Supplementary Materials

The molecular bases of the interaction between a saponin from the roots of *Gypsophila paniculata* L. and model lipid membranes

Beata Korchowiec[1,*], Jacek Korchowiec[1], Klaudia Kwiecińska[1], Reneta Gevrenova[2], Sabine Bouguet-Bonnet[3], Cheng Deng[3], Max Henry[4], Ewa Rogalska[4]

[1]*Faculty of Chemistry, Jagiellonian University, ul. Gronostajowa 2, 30-387 Krakow, Poland*

[2]*Department of Pharmacognosy, Faculty of Pharmacy, Medical University-Sofia, 2 Dunav Str., 1000 Sofia, Bulgaria*

[3]*Université de Lorraine and CNRS, UMR 7053 L2CM, F-54000 Nancy, France*

[4]*SRSMC, UMR 7565, CNRS-Université de Lorraine, Boulevard des Aiguillettes, 54506 Vandœuvre-Lès-Nancy, France*

*Corresponding author: (BK) E-mail: bkorch@chemia.uj.edu.pl; orcid.org/0000-0003-3950-7514. Telephone: +48 (12) 686 25 16. Fax: +48 (12) 686 27 50.

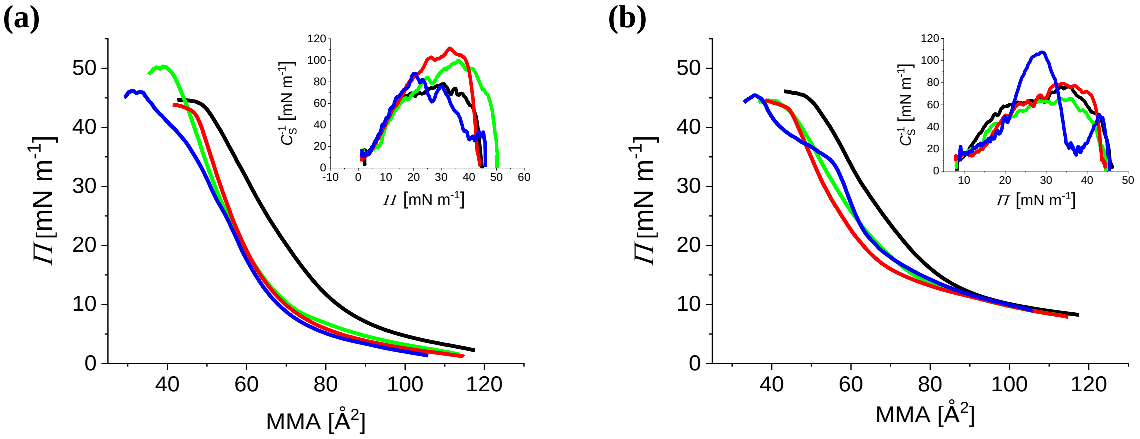

Figure S1. Compression isotherms of SM/POPC (black), SM/POPC/CHOL 10 mol% (green), SM/POPC/CHOL 30 mol% (red), and SM/POPC/CHOL 50 mol% (blue) mixed films spread on GOTCAB solution subphase: 8 (a), and 80 mg L^{-1} (b). Inset: C_S^{-1}–Π dependency. T = 20 ºC.

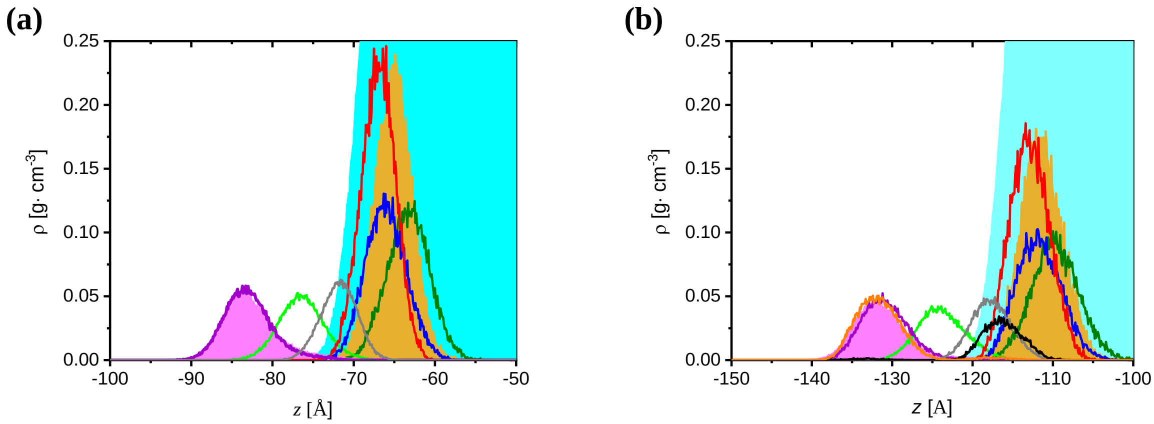

Figure S2. Partial density plots at the interface of SM/POPC (a) and SM/POPC/CHOL 30 mol% (b) monolayers. Color code: cyan area: water; magenta area: terminal CH_3 in the POPC lateral chains; violet line: terminal CH_3 in the SM lateral chains; orange line: terminal CH_3 in the CHOL lateral chain; green line: C=C in POPC; gray line: C=C in SM; orange area: PO_4 in POPC; red line: PO_4 in SM; black line: OH in CHOL; olive line: $N(CH_3)_3$ in POPC; blue line: $N(CH_3)_3$ in SM.

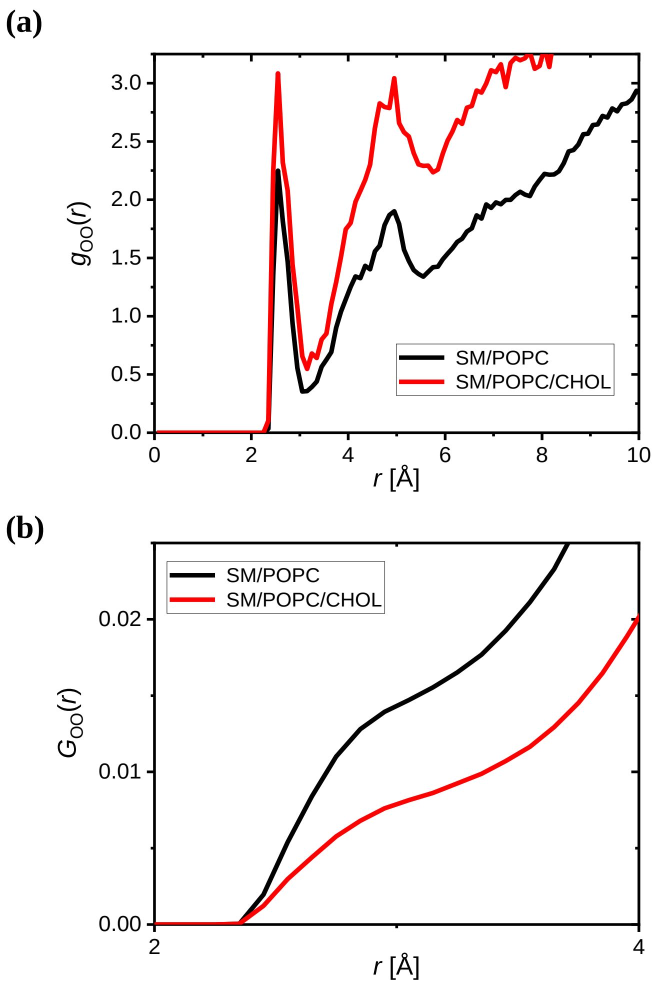

Figure S3. Radial pair distribution function, $g_{\mathrm{OO}}(r)$, (a) and integral over radial pair distribution function, $G_{\mathrm{OO}}(r)$, (b) for hydroxyl oxygen atoms of glycone moiety and oxygen atoms of PO_4 residues. The black and red curves correspond to SM/POPC and SM/POPC/CHOL, respectively.

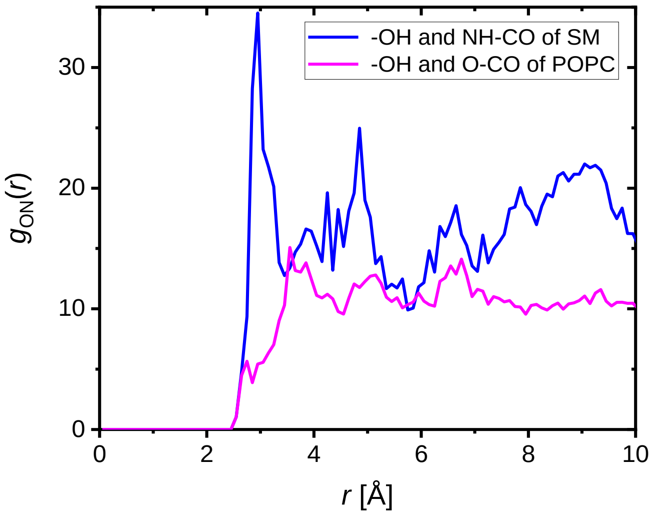

Figure S4. Radial pair distribution function between hydroxyl oxygen atoms of GOTCAB aglycone moiety and nitrogen –NH- unit of SM, $g_{\mathrm{ON}}(r)$. Only GOTCAB molecules penetrating hydrophobic part of the monolayer were considered in the calculations.

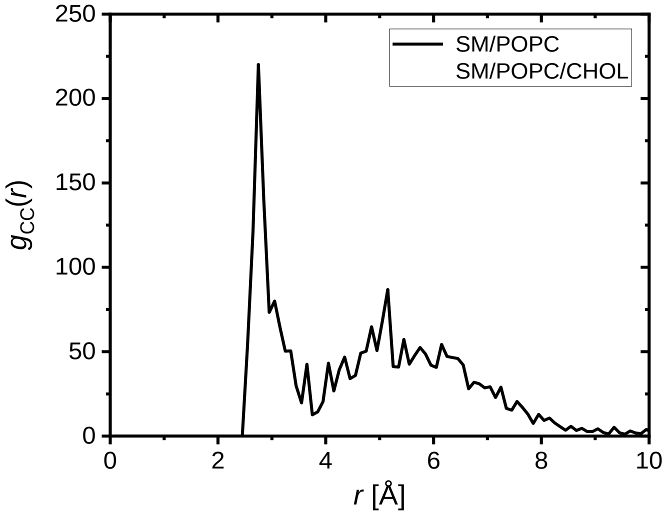

Figure S5. Radial pair distribution function between hydroxyl oxygen atoms of GOTCAB aglycone moiety and oxygen –NHCO- unit [$g_{OO}(r)$] of SM. Only GOTCAB molecules penetrating hydrophobic part of the monolayer were taken into account in the calculations.

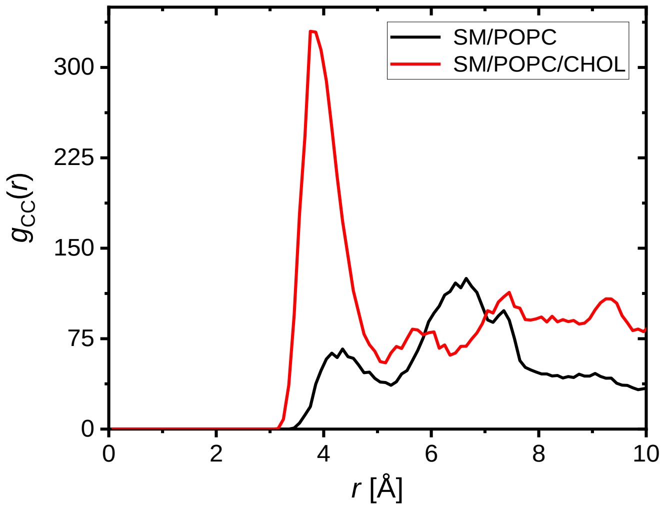

Figure S6. Radial pair distribution functions for methyl carbon atoms of aglycone moiety and methyl carbon atoms of rhamnose and fucose residues. The black and red curves correspond to SM/POPC and SM/POPC/CHOL monolayer systems, respectively.
